# Native left ventricular myocardial T_1_ spatial heterogeneity in non-ischemic dilated cardiomyopathy

**DOI:** 10.1186/1532-429X-18-S1-Q41

**Published:** 2016-01-27

**Authors:** Abyaad Kashem, Ravi V Shah, Shingo Kato, Steven Bellm, Sébastien Roujol, Tamer Basha, Jihye Jang, Warren J Manning, Reza Nezafat

**Affiliations:** 1 grid.239395.70000000090118547Department of Medicine (Cardiovascular Division), Beth Israel Deaconess Medical Center, Boston, MA USA; 2 grid.239395.70000000090118547Department of Radiology, Beth Israel Deaconess Medical Center, Boston, MA USA

## Background

Myocardial fibrosis is involved in the pathology of non-ischemic dilated cardiomyopathy (NICM). Recently, the application of native (non-contrast) myocardial T_1_ measurement has been proposed as an imaging biomarker of cardiac remodeling. However, spatial heterogeneity in T_1_ measurements has been observed across different segments and slices. Furthermore, T_1_ values measured with current T_1_ mapping sequences are influenced by myocardial T_2_ values. The objective of this study was to 1) assess the spatial heterogeneity of T_1_ measurements across different segments and slices in healthy subjects and patients, and 2) determine the association of native T_1_ with myocardial structure and function.

## Methods

We prospectively studied 39 NICM patients (LVEF≤50% without evidence of prior infarction by CMR) and 30 subjects with normal LV systolic function without known cardiovascular disease. CMR was performed using a 1.5-T MRI scanner (Philips Achieva). Native T_1_ mapping was performed using slice-interleaved T_1_ mapping sequence (STONE) [1]. T_2_ mapping was performed using slice-interleaved T_2_ mapping [2]. Voxel-wise T_1_ and T_2_ were estimated using a 2-parameter and 3-parameter model [3]. All images were corrected for motion [4]. T_1,_ T_2,_ and extra-cellular volume (ECV) measurements were measured using a 16 segments AHA model across the base, mid, and apical LV.

## Results

**Figure 1:**
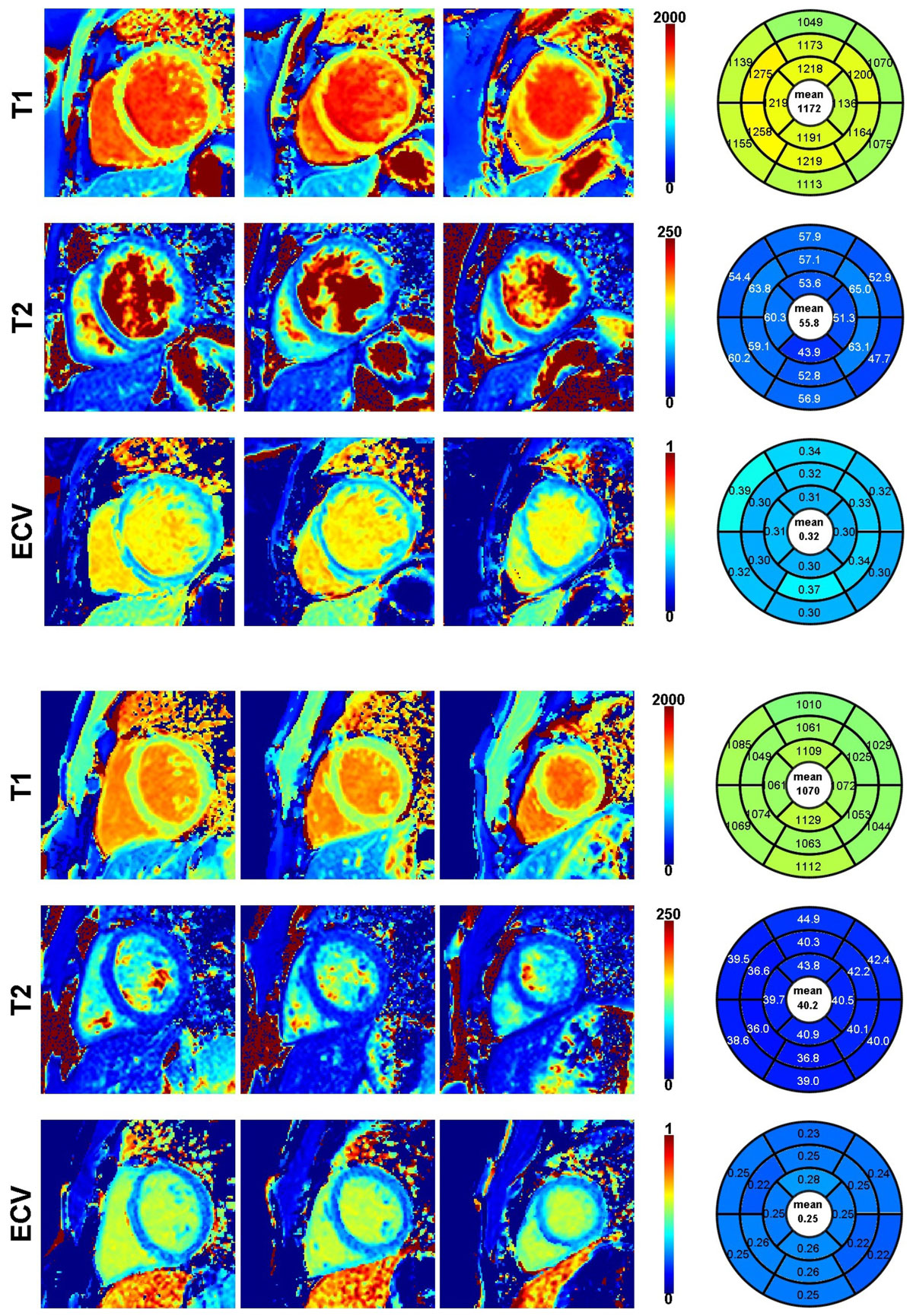
**(A) Images from a 47-year-old woman with a NICM with moderate reduction in LV function (LV ejection fraction 30%)**. (B) Images from a 39-year-old heathy male (LV ejection fraction 61%).

**Figure 2:**
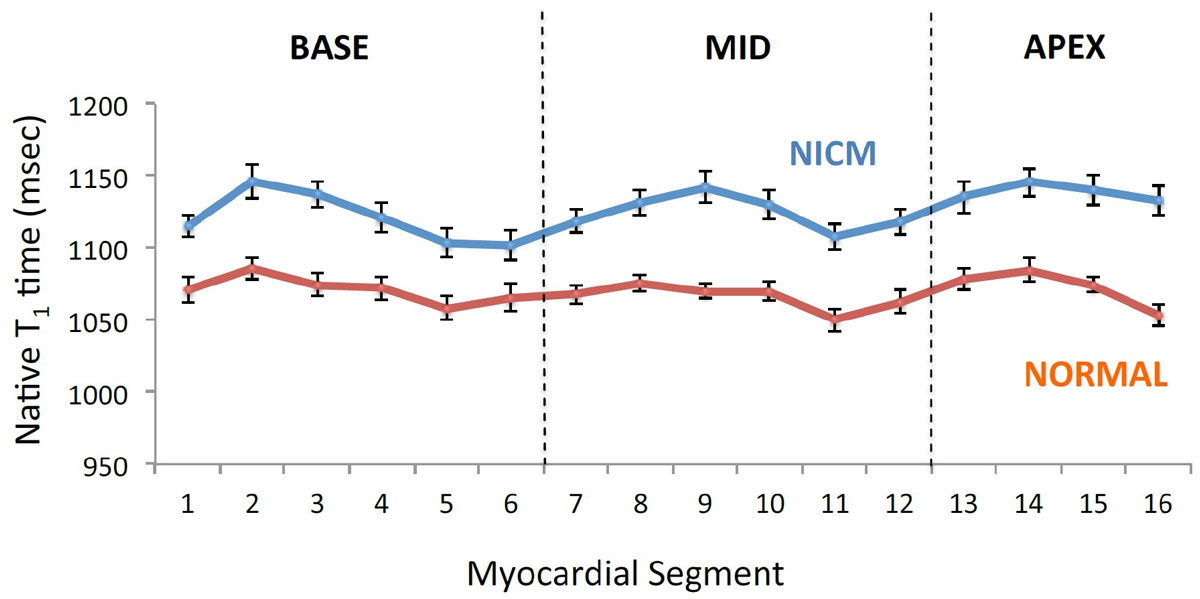
**Variability in segmental native T**_**1**_ **across all 16 myocardial segments in individuals with normal LV function (red) and study participants with NICM (blue)**. The error bars represent standard error of the mean.

NICM participants (57 ± 15 years) were predominantly male (74%). By design, all had a reduced LV ejection fraction (mean LVEF 34 ± 10%). Figure 1 shows native T1, T2, and ECV in two patients with a NICM. Parametric maps to the right in each panel demonstrate full ventricular coverage. The regional distribution of native myocardial T_1_ was similar in patients with and without NICM[RN1], as shown in Figure 2. Relative to subjects without NICM, subjects with NICM had a higher native T_1_ (1131 ± 51 vs. 1070 ± 28 msec; p < 0.0001), a higher ECV (0.28 ± 0.04 vs. 0.25 ± 0.02, P = 0.001) and a longer myocardial T_2_ (52 ± 8 vs. 47 ± 5 msec; P = 0.01). After multivariable adjustment, a lower global native T_1_ time was associated with a higher LVEF (b = -0.59, P = 0.0003), higher right ventricular ejection fraction (b = -0.47, P = 0.006), and lower left atrial volume index (b = 0.51, P = 0.001).

## Conclusions

In NICM, native myocardial T_1_ is elevated in a homogeneous manner, suggesting a global (not regional) abnormality in myocardial tissue composition. This low variability is similar between healthy and NICM patients across different segments. For subjects with NICM, native T_1_ is associated with biventricular systolic function and left atrial volume, and may represent a non-contrast marker of tissue remodeling in this cohort.
